# Soybean Protein Hydrolysate Enhances Growth and Freeze-Drying Survival of *Bifidobacterium breve* and *Bifidobacterium longum* Strains

**DOI:** 10.3390/foods14234071

**Published:** 2025-11-27

**Authors:** Lanyan Huang, Xinyu Zhao, Qingping Wu, Weipeng Guo, Ning Yang, Yue Fan, Ying Zhang, Ying Li, Xinqiang Xie, Moutong Chen

**Affiliations:** 1 Guangzhou Institute of Chemistry, Chinese Academy of Sciences, Guangzhou 510650, China; huanglanyannn@163.com; 2 State Key Laboratory of Applied Microbiology Southern China, Guangdong Provincial Key Laboratory of Microbial Safety and Health, National Health Commission Science and Technology Innovation Platform for Nutrition and Safety of Microbial Food, Key Laboratory of Big Data Technologies for Food Microbiological Safety, State Administration for Market Regulation, Institute of Microbiology, Guangdong Academy of Sciences, Guangzhou 510070, Chinaliying@gdim.cn (Y.L.);; 3 Food and Drug Laboratory, Guangdong Detection Center of Microbiology, Guangzhou 510070, China; 4 Guangdong Huankai Biotechnology Co., Ltd., Guangzhou 510663, China; 5 University of Chinese Academy of Sciences, Beijing 100049, China

**Keywords:** probiotics, soybean protein hydrolysates, peptides, *Bifidobacterium*, growth promotion, bacterial stability, potential prebiotics

## Abstract

The study proposed a strategy using soybean protein hydrolysate (SPH) as the sole nitrogen source for promoting the proliferation and freeze-drying survival of *Bifidobacterium breve* and *Bifidobacterium longum* strains. High proportions of SPH replacing traditional nitrogen sources in MRSL significantly enhanced bacterial viable cell counts and OD_600_ values. The small peptides (<3 kDa) and hydrophilic amino acid residues in SPH are considered to be the key factors for promoting bacterial growth. The exclusive use of SPH (100%-SPH) as the nitrogen source induced a morphological change in *B. breve* 1206 with the Y-shape transformation into smaller rod-shaped cells, while *B. longum* 070103 and 050101 became shorter rods. Cells with these morphological changes could more effectively maintain cell membrane integrity in an acidic condition and during the freeze-drying process. Consequently, MSPH improved cell viability and freeze-drying survival for *B. breve* 1206 in PBS and 10% skim milk compared to MRSL. It also significantly increased viable cell counts and the ability to survive freeze-drying for functional *B. longum* strains 070103 and 050101 in 10% skim milk, with survival rates increasing by 16.2% and 43.1%, respectively. These results showed the applicability of SPH in the industrial-scale cultivation of functional *Bifidobacterium* strains. It also provides new insights into soybean-derived nitrogen sources that can affect amino acid composition and bacterial morphology to enhance probiotic stability. This study supported the application of soybean peptides as the sole nitrogen source for producing high-viability probiotics and the potential prebiotic with health benefits.

## 1. Introduction

The probiotics market has witnessed remarkable expansion in recent years. In 2023, the market had a value of USD 87.70 billion and is expected to increase at a compound annual growth rate of 14.1% before 2030 [1]. This growth is largely due to the increasing application of probiotics to promote gut health, enhance immunity, and support digestion, particularly in functional foods, beverages, and dietary supplements [2,3]. Probiotics are typically found in *Bifidobacterium* or *Lactobacillus*. They are generally recognized for their fastidiousness regarding nutritional requirements and culture conditions. Moreover, both *Lactobacillus* and *Bifidobacterium* strains grow slowly, and their viability declines rapidly over production, transportation, and storage [4]. This poses a major challenge for their commercialization. Previous study suggests that probiotic foods need to contain no less than 10^6^~10^7^ CFU/g to be effective [5]. Improving fermentation efficiency and survival during processing and storage to obtain the long-term viability and stability of probiotic products is very important.

Prebiotics, mainly oligosaccharides, have been widely used for supporting the growth of probiotics and improving the health of the host [6,7]. Recently, protein hydrolysates and their derived peptides have gained increasing attention as novel prebiotics [8]. They provide essential amino acids and peptides that can support probiotics’ growth and metabolism [9,10]. Nevertheless, traditional animal protein sources have drawbacks with high costs, the potential risk of disease transmission (such as bovine spongiform encephalopathy), and limitations on use by religious beliefs and vegetarianism [11,12]. These factors are unfavorable for industrial production, limit the accessibility to certain populations, and thus hinder the further popularization of probiotic products. In contrast, plant protein sources have several advantages as nitrogen sources, such as being more environmentally friendly, sustainable, and free from religious and cultural restrictions. Soybean protein, with its rich nutritional components, is an ideal alternative nitrogen source. Research has demonstrated that soybean-derived peptides can enhance the proliferation and metabolic processes of probiotic strains belonging to the genera *Lactobacillus* and *Bifidobacterium* [13,14]. Current studies focused on the effects of soybean-derived peptides on their role in simulating gastrointestinal digestion and their application in dietary supplements [15,16]. Soy-based products fermented with probiotic bacteria, such as soy milk and soybean sprout yogurt-like products, have also been investigated for health-related benefits, including improvements in blood parameters, regulation of body weight, and increased formation of bioactive γ-aminobutyric acid [17,18]. Nevertheless, limited information about the promoting effects of soybean peptides as the sole nitrogen source in the high-density fermentation process of probiotics in vitro is available.

In addition to fermentation efficiency, the storage method of probiotics also directly determines their final survival rate and effectiveness. Freeze-drying is commonly employed in the preparation of probiotic powders. During this process, ice crystals formed and the dehydrated solute concentration changed, leading to cell membrane rupture and internal structure damage, greatly reducing the strain’s survival [19,20]. The viability of probiotics in the freeze-drying process could be improved by modifying culture conditions, selecting appropriate protectants, and optimizing parameters [21]. A previous study revealed that adding aspartic acid to the medium reduced cell structure and DNA damage in strains, thereby improving lyophilized survival [22]. Additionally, another study showed that more robust strains with shorter cell morphology could be obtained by optimizing the medium, thus improving their stability during lyophilization and storage [23].

Bifidobacteria strains are widely recognized as probiotics with significant gut health benefits [24]. There are significant differences in the distribution of proteolytic systems among different *Bifidobacterium* strains, including cell-envelope proteinases (CEPs), oligopeptide transport systems, and intracellular peptidases. These variations determine their ability to utilize protein hydrolysates and thus affect their growth rate [25,26]. In particular, *Bifidobacterium* is a typical genus with amino acid nutritional deficiencies. In addition, few CEPs with activity in *Bifidobacterium* are found, hence increasing the difficulty of their proliferation in dairy fermentation, such as in milk [27,28,29].

Although the growth-promoting effects of soybean peptides on probiotics have been reported, few studies have explored their use as the sole nitrogen source to promote the growth of probiotics and further improve survival rate during subsequent lyophilization. In addition, animal-derived protein sources have limitations in terms of cost, safety, and acceptability, which has led to growing attention on plant-derived protein hydrolysates as alternative nitrogen sources for probiotic fermentation. Therefore, this study aims (i) to explore soybean protein hydrolysates (SPH) as an alternative nitrogen source for *Bifidobacterium breve* 1206 by analyzing the effects of SPH on its growth, morphology, and freeze-drying survival, and (ii) to further evaluate the applicability of this strategy for the functional *Bifidobacterium longum* strains (070103 and 050101). These findings are expected to support for the use of soybean-derived peptides in high-density proliferation, lyophilization preservation, and potential prebiotic applications for *Bifidobacterium* strains.

## 2. Materials and Methods

### 2.1. Extraction of Soy Protein Isolate

The low-temperature soybean meal (Yuwang Ecological Food Industry Co., Ltd., Dezhou, China) was utilized as the raw material, with a crude protein content of 48.6% (conversion factor is 6.25) measured using the Kjeldahl nitrogen method [30]. The alkaline extraction-isoelectric precipitation process applied by Stone et al. [31], with small adjustments, was employed to obtain soy protein isolate (SPI). In brief, 100 g of soybean meal was mixed with deionized water at a ratio of 1:10 (*w*/*v*), and the pH was adjusted to 8.5. The suspension was shaken at 55 °C for 2 h and filtered with gauze before centrifugation. Then the supernatant was modified to pH 4.5 (approximately at the isoelectric point of SPI), followed by centrifugation to separate the insoluble precipitate. The precipitate was rinsed three times using deionized water and the pH was set to 7.0. The extracted proteins were lyophilized and stored in sealed containers for further study. The protein content of SPI powder was calculated to be 92%.

### 2.2. Enzymatic Hydrolysis of Soy Protein Isolate

A 5% water suspension of SPI was preheated before enzymatic hydrolysis to prepare the soy protein hydrolysates (SPH). Proteolysis was performed at 55 °C and pH 8.5 using 2% (*w*/*v*) Alcalase 2.4 L FG (Novozymes Biotechnology Co., Ltd., Tianjin, China) for 6 h, as previously described [32], with minor modifications. The pH of the suspension was maintained at 8.5 consistently during the hydrolysis process. Different samples were prepared with a hydrolysis duration at each 1 h interval within 6 h, respectively. The hydrolysates were boiled to inactivate the enzyme, followed by centrifugation to separate the supernatant and precipitate. Finally, the supernatant was concentrated and kept at −20 °C for further analysis. The nitrogen content of hydrolysates was also measured by the Kjeldahl nitrogen method and a conversion factor multiplying total nitrogen by 6.25 (N × 6.25) to calculate the protein content.

### 2.3. Characterization of SPH

#### 2.3.1. Degree of Hydrolysis

The degree of hydrolysis (DH) of the enzymatic hydrolysis products at different time points was analyzed by the OPA method established by Nielsen, Petersen, and Dambmann [33]. In detail, 0.4 mL of diluted SPH was blended with 3 mL of OPA solution and reacted for 2 min. Immediately, a UV-5880 spectrophotometer (Unico, Shanghai, China) was applied to record the absorbance at 340 nm. Serine (0.9516 mmol/L) served as the standard solution and deionized water as blank. The DH was calculated based on the equations below.

(1) $$Serine-{NH}_{2}=\frac{{OD}_{sample}-{OD}_{blank}}{{OD}_{standard}-{OD}_{blank}}\times \frac{\left(0.9516\times V\times 100\right)}{\left(X\times P\right)}$$

(2) $$h=\frac{\left(Serine-{NH}_{2}\right)-\beta }{\alpha }$$

(3) $$DH(\%)=\frac{h}{h_{tot}}\times 100$$

where *V* is the sample volume (L), *X* is the sample weight (g), and *P* is the protein content (%), *α* is 0.970, *β* is 0.342, *h* is the number of hydrolyzed peptide bonds, and *h_tot_* is 7.8 for SPI.

#### 2.3.2. SDS-PAGE

The soybean protein isolate and its hydrolysates were analyzed by SDS-PAGE with 5% stacking gels and 12% resolving gels using the Mini-PROTEAN Tetra Cell (Bio-Rad, Hercules, CA, USA) as described by Cui et al. [34]. The SDS-PAGE preparation kit was provided by Shanghai Sangon Biotech Co., Ltd. (Shanghai, China). The 20 μL samples of the SPI dispersion (20 mg/mL) and each SPH sample were blended with 5 μL of 5 × loading buffer (Solarbio Science & Technology Co., Ltd., Beijing, China) and boiled for 10 min. Each 10 μL of the mixed sample solution and molecular markers with 10–180 kDa (Beijing Lablead Trading Co., Ltd., Beijing, China) was loaded. Initially, the voltage for electrophoresis was set at 80 V and subsequently increased to 120 V as the sample entered the resolving gel. The resulting gels were dyed using Coomassie Brilliant Blue R-250 (Shanghai Sangon Biotech Co., Ltd., Shanghai, China) and decolorized several times with a destaining solution (acetic acid/ethanol/water in a volume ratio of 1:4:5) until the image became clear.

#### 2.3.3. Molecular Weight Distribution

The ultra-high performance liquid chromatograph (UHPLC) Nexera XR System (Shimadzu Scientific Instruments, Kyoto, Japan) was used to analyze the molecular weight distribution of SPH following a modified protocol by Coscueta et al. [35]. Prior to HPLC analysis, a 0.22 μm filter membrane was employed for filtering SPH samples. The sample was eluted on an AdvanceBio SEC 300 Å column (2.7 μm, 300 × 7.8 mm, Agilent Technologies, Santa Clara, CA, USA) with a flow rate of 0.6 mL/min and an injection of 20 μL. Protein standards (Shanghai Yuanye Science & Technology Co., Ltd., Shanghai, China), including ovalbumin (45,000 Da), insulin (5777 Da), oxidized glutathione (612 Da), Gly-Gly-Tyr-Arg (451 Da), and 2′-deoxycytidine (227 Da), were used to generate a calibration curve for molecular weight determination of the eluted peaks.

#### 2.3.4. Trichloroacetic Acid–Soluble Protein Content

The trichloroacetic acid (TCA)-soluble protein content was determined and calculated according to the method of Henn et al. [36]. TCA was added to the inactivated hydrolysates to make the final concentration of 10% (*w*/*v*) to precipitate proteins and high-molecular-weight peptides. The supernatant was obtained by centrifugation, and the soluble nitrogen was determined by the Kjeldahl method [30].

### 2.4. Amino Acid Composition Analysis

The composition of amino acids was analyzed based on the method of Wu et al. [37] with minor modifications. Briefly, 1 mL of supernatant and 10 mL of 6 mol/L HCl were added to the digestion tube. Then it was sealed, and hydrolysis was conducted in a 110 °C oven for 22 h. The solution was shifted to a 50 mL volumetric flask and diluted with ultrapure water. Subsequently, 2 mL of diluted solution was dried and then redissolved with 0.02 mol/L HCl. The amino acid concentration of the solution was determined by an automatic amino acid analyzer (L-8900, Hitachi Ltd., Tokyo, Japan).

### 2.5. Microorganisms and Culture Conditions

*Bifidobacterium breve* 1206 and *Bifidobacterium longum* (strains 070103 and 050101) were provided by Guangdong Microbial Culture Collection Center (Guangzhou, China). The strains stored at −80 °C were cultured anaerobically at 37 °C for 48 h in commercial deMan Rogosa, Sharpe (MRS), medium (Guangdong Huankai Microbial Science & Technology Co., Ltd., Guangzhou, China) added with 0.05% (*w*/*v*) L-cysteine. After being activated three times in succession, bacterial solutions incubated for 24 h were used for further experiments.

Furthermore, the MRS medium supplemented with L-cysteine (marked as MRSL) for the control group in the growth promotion experiment was self-prepared. The control MRSL medium was composed of these ingredients: tryptone (10 g/L), beef extract (5 g/L), yeast extract (4 g/L), ammonium citrate (2 g/L), sodium acetate (5 g/L), MgSO_4_∙7H_2_O (0.2 g/L), MnSO_4_ (0.05 g/L), K_2_HPO_4_ (2 g/L), glucose (20 g/L), Tween 80 (1 g/L), and L-cysteine (0.5 g/L). Tryptone, beef extract, and yeast extract, which were provided as nitrogen sources in MRSL medium, were replaced with soybean protein hydrolysates in different ratios (25%, 50%, 75%, and 100%), referred to as the 25%-SPH, 50%-SPH, 75%-SPH, and 100%-SPH groups (MSPH), respectively. The nitrogen level is equal in all media. All prepared media were adjusted to pH 6.2 before autoclaving at 121 °C for 15 min.

To determine whether it is the peptide or the free amino acid in MSPH that plays a role in the growth-promoting effect, an amino acid medium was prepared. The prepared amino acids served as the nitrogen source, and the other components were the same as those in MRSL. The amino acid nitrogen source was prepared based on the amino acid composition of the measured MSPH. At the same time, the hydrophilic amino acid culture medium was also prepared according to the proportion of MSPH. The total nitrogen content of both media was the same as that of MRSL. Finally, the pH was adjusted to 6.2, and the medium was sterilized before use.

### 2.6. Determination of Growth of Bifidobacteria Strains In Vitro

The bacterial cells cultured for 24 h were centrifuged at 5000 rpm for 10 min at 4 °C, and then the bacterial pellet was rinsed with phosphate-buffered saline (PBS, pH 7.0) to remove culture media. The bacteria were resuspended in sterile saline water to an OD_600_ nm of 0.8, then inoculated 3% (*v*/*v*) into the control and experimental medium. And the initial concentration was about 10^7^ CFU/mL.

The total number of viable bacteria was counted by the plate count method. The fermentation samples at 24 h were serially diluted in sterile saline and spread on commercial MRS agar supplemented with 0.05% (*w*/*v*) L-cysteine. Plates were incubated at 37 °C for 48 h under anaerobic conditions before colony counting. In order to determine the growth curve, the inoculated bacterial solution was transferred into a 96-well plate and incubated anaerobically (10% CO_2_ and 90% N_2_) at 37 °C for 24 h in the Cytation 5 multimode plate reader (BioTek, Winooski, VT, USA). The OD value at 600 nm was automatically measured at 30 min interval.

The growth generation time was calculated by fitting the slope of the logarithmic phase of the probiotic growth curve, as shown in Equation (4).

(4) $$g=\frac{ln2}{\mu }$$

where *g* represents the doubling time and *μ* is the slope of the logarithmic phase.

Under the same conditions, *B. breve* 1206 was cultured in MRSL and 25%~100%-SPH in conical flasks. The pH of samples at different time points was measured using a pH meter. For *B. longum* 070103 and 050101 strains, the total number of viable bacteria was determined under MRSL and MSPH conditions.

### 2.7. Effects of SPH as Sole Nitrogen on the Freeze-Drying Survival Rate

*B. breve* 1206 was cultured in MRSL and MSPH (100%-SPH) medium for 24 h. The collected bacteria were thoroughly mixed with phosphate-buffered saline (PBS) and 10% skim milk as protectant at a ratio of 1:2 (*m*/*v*), respectively. The same volume of bacterial suspension was frozen at −80 °C for 4 h and then vacuum freeze-dried for 48 h. The viable cell number before and after lyophilization was evaluated by the plate counting method. And 10% skim milk was used as the protectant for the functional strains *B. longum* 070103 and 050101, and all other conditions were the same as those for *B. breve* 1206.

The freeze-drying survival rate was calculated as follows.

$$Survival\;rate\;\left(\%\right)=\frac{Viable\;cell\;number\;after\;lyophilization}{Viable\;cell\;number\;before\;lyophilization}\times 100$$

### 2.8. Scanning Electron Microscopy (SEM) Analysis

Scanning electron microscopy (SEM) was used to characterize the cell morphology of *Bifidobacterium strains* cultured in MRSL and MSPH. Sample pretreatment was performed as previously described [38]. After 24 h of incubation, bacterial cells were collected and fixed overnight with 2.5% glutaraldehyde at 4 °C. The samples were then dehydrated through a graded series of ethanol (30%, 50%, 70%, 80%, 90%, and 100%), followed by treatment with tert-butanol and freeze-dried. The freeze-dried bacterial samples, after adding protective agents, required no pretreatment. All the bacterial samples were coated with gold before observation.

### 2.9. Confocal Laser Scanning Microscopy Imaging

The viability of *B. breve* 1206 during incubation in the control and the soybean protein hydrolysate group was assessed using the LIVE/DEAD BacLight Bacterial Viability Kit (Thermo Fisher Scientific, Eugene, OR, USA). Briefly, equal volumes of SYTO 9 (green fluorescence) and propidium iodide (PI, red fluorescence) were mixed. A total of 997 µL of suspended cells was supplemented with 3 µL of the mixed staining solution and incubated in the dark environment for 15 min. Fluorescence was subsequently observed using a confocal laser scanning microscope (CLSM, Zeiss LSM 800, Carl Zeiss AG, Oberkochen, Germany) with 63× magnification objective and with 488 nm argon laser and a 561 nm laser for simultaneous imaging of green and red fluorescence.

### 2.10. Statistical Analysis

All experiments were performed for three replications, and the results were presented as mean ± standard deviation (mean ± SD). Statistical analysis was conducted using GraphPad Prism 8.0.2 (GraphPad Software Inc., San Diego, CA, USA) for *t*-test and SPSS 26.0 (IBM Corp., Armonk, NY, USA) for one-way analysis of variance (ANOVA) followed by Tukey’s post hoc test. Differences were considered statistically significant at *p* < 0.05.

## 3. Results and Discussion

### 3.1. Effects of Enzymatic Hydrolysis on Protein Degradation

#### 3.1.1. Degree of Hydrolysis

**Figure 1:**
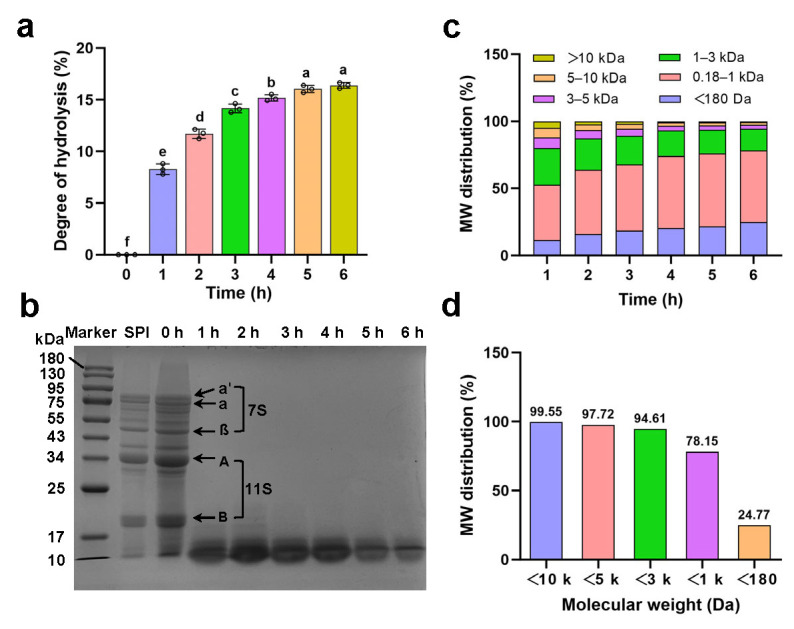
(**a**) Degree of hydrolysis of soy protein isolate by Alcalase from 0 to 6 h; (**b**) SDS-PAGE pattern of soy protein isolates and Alcalase-produced protein hydrolysates from 0 to 6 h. M: molecular weight marker; SPI: soy protein isolate; 0–6: Alcalase hydrolysate of 0–6 h; (**c**) the molecular weight (MW) distribution of hydrolysates at 1–6 h; (**d**) the MW distribution of hydrolysates at 6 h. Different letters indicate significant differences at *p* < 0.05 (SPSS analysis).

As the hydrolysis time increased, the degree of hydrolysis of SPH gradually improved (Figure 1a). The degrees of hydrolysis (DH) from 1 h to 6 h were 8.28%, 11.70%, 14.17%, 15.17%, 16.04%, and 16.39%, respectively. The hydrolysis rate increased rapidly during the first three hours, while there was no apparent change in the degree of hydrolysis during the fifth and sixth hours, indicating that hydrolysis had reached a stable state. The hydrolysate for 6 h was subsequently used as the nitrogen source for medium preparation.

#### 3.1.2. Molecular Weight Distribution

As shown in Figure 1b, the SDS-PAGE results illustrated the molecular weight distribution during the hydrolysis process of SPI. After hydrolysis for 1 h, the primary 7S and 11S proteins had been hydrolyzed, leading to the disappearance of multiple bands within the range of 17–95 kDa, with a molecular weight distribution below 17 kDa. This result suggested that Alcalase 2.4 L FG achieved efficient hydrolysis of SPI. Additionally, during the 1–6 h period, the hydrolysate with molecular weight between 10 and 17 kDa and below 10 kDa gradually diminished with the increase in hydrolysis time, indicating that further hydrolysis of protein could produce smaller peptides.

HPLC analysis was performed to further assess the molecular weight distribution and the proportion of small peptides of the hydrolysates at different hydrolysis times. As shown in Figure 1c, during the 1–6 h period, the proportions of peptides greater than 10 kDa, as well as those in the 5–10 kDa, 3–5 kDa, and 1–3 kDa ranges, all decreased with the increasing of hydrolysis time. Conversely, the proportions of peptides between 180 and 1000 Da and below 180 Da gradually increased. Peptides below 180 Da are generally classified as free amino acids. This indicates that the proportion of peptides below 1000 Da and free amino acids increases with prolonged hydrolysis time.

Previous studies have demonstrated that small peptides derived from various sources can effectively promote the proliferation of probiotics, and most of these peptides possess molecular weights below 3000 Da, particularly those below 1000 Da [8]. Zhao et al. [39] reported that peptides from SPH with molecular weights below 3000 Da exhibit the highest growth-stimulating activity for *Streptococcus thermophilus*. Additionally, casein peptides below 1000 Da significantly enhance the growth of commonly found dairy-related bifidobacteria [40]. In this study, the proportion of peptides below 3 kDa reached 94.61% after 6 h of hydrolysis, and the proportion below 1 kDa was 78.15% (Figure 1d). This result suggested that SPH mainly consists of low-molecular-weight peptides, indicating its potential as a source of growth-promoting peptides for probiotics.

### 3.2. Effects of Hydrolysis Time and Nitrogen Substitution Ratio on the Growth of B. breve 1206

**Figure 2:**
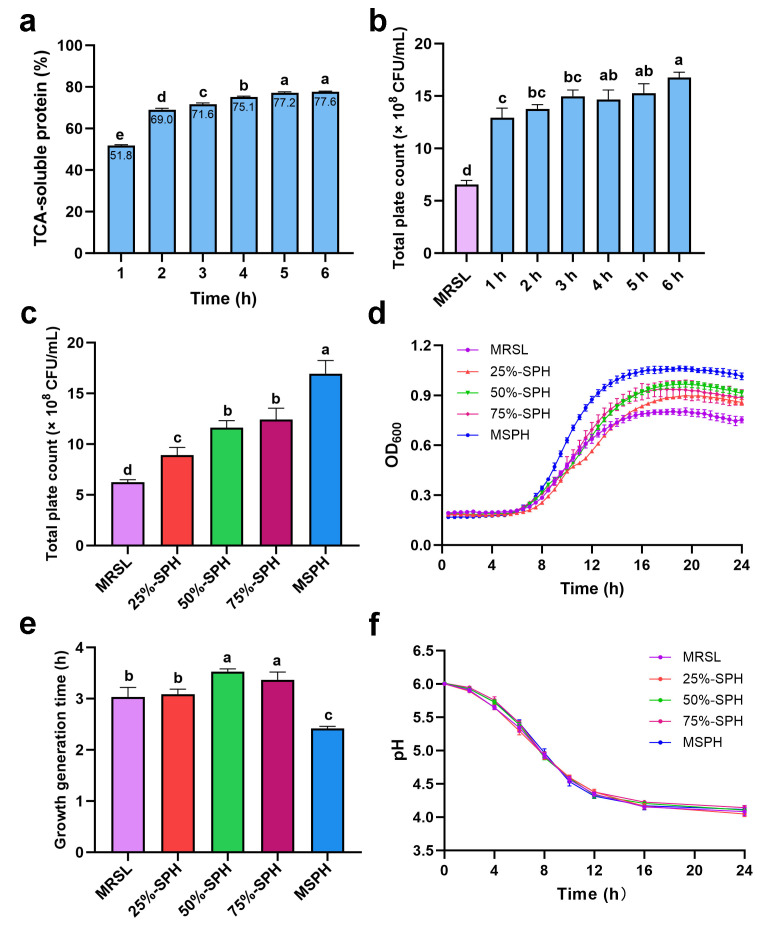
(**a**) Trichloroacetic acid (TCA)-soluble protein contents in SPH samples hydrolyzed for 1 to 6 h; (**b**) total plate count of *B. breve* 1206 cultured in MRSL and different SPHs (1 to 6 h) as sole nitrogen source for 24 h; (**c**) growth curves; (**d**) total plate count at 24 h; (**e**) growth generation time; and (**f**) pH changes in *B. breve* 1206 cultured of different medium groups (MRSL, 25%-SPH, 50%-SPH, 75%-SPH, and 100%-SPH (MSPH)) prepared by soy protein isolate hydrolysates. Different letters among groups indicate significant differences at *p* < 0.05 (SPSS analysis).

To determine the optimal enzymatic hydrolysis time, this study systematically evaluated the changes in TCA-soluble protein content during 1 to 6 h of hydrolysis and the growth-promoting effect of the products on *B. breve* 1206. As shown in Figure 2a, the TCA-soluble protein content of the hydrolysates increased significantly from 51.8% to 75.1% between 1 and 4 h of hydrolysis. After 4 h, the rate of increase slowed down. At 5 and 6 h, it was 77.2% and 77.6%, respectively, with no significant difference between them. These results are generally consistent with the degree of hydrolysis, indicating that hydrolysis time has a major impact on yield of soluble protein.

Meanwhile, growth promotion of the 1–6 h hydrolysates (Figure 2b) showed that the viable cell counts for all SPH samples were significantly higher than those of the control group MRSL. The 6 h hydrolysate resulted in the highest viable count, while the 1 h sample was the lowest. Although significant differences were observed between the 1, 2, and 3 h hydrolysates compared to the 6 h sample, no statistical differences in growth-promoting effect were found for 1 to 3 h, 2 to 5 h, and 4 to 6 h. The peptide molecular weight distribution in Figure 1c further supports this observation, showing no major changes after 3 h. This also suggests that the molecular weight distribution of peptides is not the only factor affecting growth-promoting activity. Differences in activity are likely also related to the abundance of specific bioactive peptides and their complex interactions within the hydrolysates.

In this study, to better address the research questions, the 6 h hydrolysates were used for further experiments due to their best growth effect and highest TCA-soluble protein content. However, for industrial production, it is necessary to consider the balance between the TCA protein yield, the cost of hydrolysis time, and functional characteristics. The results above indicate that once key functional peptides are released during the initial stage (first 3 h) of hydrolysis, further extending the hydrolysis time provides limited and uneconomical gains in activity.

For assessing the potential of SPH as a prebiotic for bifidobacteria, the protein hydrolysates prepared for 6 h were used as nitrogen sources to replace those in MRSL at different ratios for the in vitro culture of *B. breve* 1206. As shown in Figure 2c, the total colony count at 24 h was consistent with the growth trend observed in the growth curve (Figure 2d). The colony count of 25%-SPH (8.90 × 10^8^ CFU/mL) was significantly (*p* < 0.05) higher than that of MRSL (6.23 × 10^8^ CFU/mL). A noticeable growth in viable cell counts by one order of magnitude relative to MRSL occurred when SPH exceeded 50%, with 50%-SPH, 75%-SPH and 100%-SPH (MSPH) reaching 1.16 × 10^9^ CFU/mL, 1.24 × 10^9^ CFU/mL and 1.69 × 10^9^ CFU/mL, respectively. Especially MSPH (100%-SPH), showing the highest number of viable cells, exhibited a significant (*p* < 0.05) increase compared to other groups. Overall, the total colony count increased with the rising proportion of SPH in the nitrogen source and consistently exceeded that of MRSL. After 24 h, the viable cell counts for 25%-SPH, 50%-SPH, 75%-SPH, and MSPH were 1.43, 1.86, 1.99, and 2.71 times higher than that of MRSL, respectively.

As shown in Figure 2d, the cell concentration of each medium containing SPH as a nitrogen source substitute was higher than that of MRSL with the same nitrogen levels. In the stationary phase, the value of OD_600_ increased with higher SPH replacement levels from 25% to 100%, and the highest value of OD_600_ was observed in the MSPH (100%-SPH) group. Figure 2e shows that the growth generation time increased from 3.09 h at 25% SPH to 3.53 h at 50% SPH, then decreased to 3.42 h at 75% SPH and 2.41 h at MSPH (100% SPH). The growth time for 50% and 75%-SPH was significantly (*p* < 0.05) higher than the 3.03 h of MRSL, while MSPH exhibited a notably (*p* < 0.05) shorter time compared to that of MRSL.

Acid production is a key growth characteristic of probiotics. With the decrease in pH, the growth of harmful bacteria could be inhibited in the culture environment. Figure 2f demonstrated the relationship between pH and acid production of MRSL and different proportions of SPH during 24 h culture. No significant differences in pH value were observed among all the groups. All the initial pH values were approximately 6.0 (dropping from 6.2 to 6.0 after sterilization). During fermentation, the pH of the culture decreased rapidly in 12 h, finally reaching about 4.0 after 24 h. These results showed that a nitrogen-source-substituted medium with different proportions of SPH had no significant effect on the acid production of *B. breve* 1206. As a whole-plant nitrogen source, MSPH can serve as the sole nitrogen source to facilitate the growth of *B. breve* 1206.

It is suggested that SPH significantly enhances the growth rate of *B. breve* 1206, potentially due to the high proportion (94.61%) of peptides below 3 kDa present in SPH. Previous studies reported that low-molecular-weight soybean peptides could significantly promote the growth of probiotics like *Lactobacillus acidophilus* JCM1132 [13], *Limosilactobacillus reuteri* LR08 [16], and *Bifidobacterium animalis* JCM1190 [14]. Specific peptides in the hydrolysates may also supply essential amino acids for *B. breve* 1206, offering a nitrogen source superior to the mixed nitrogen source in MRSL, including beef extract, yeast extract, and tryptone, thereby enhancing its growth more effectively.

### 3.3. Amino Acid Compositions of MRSL and MSPH

**Figure 3:**
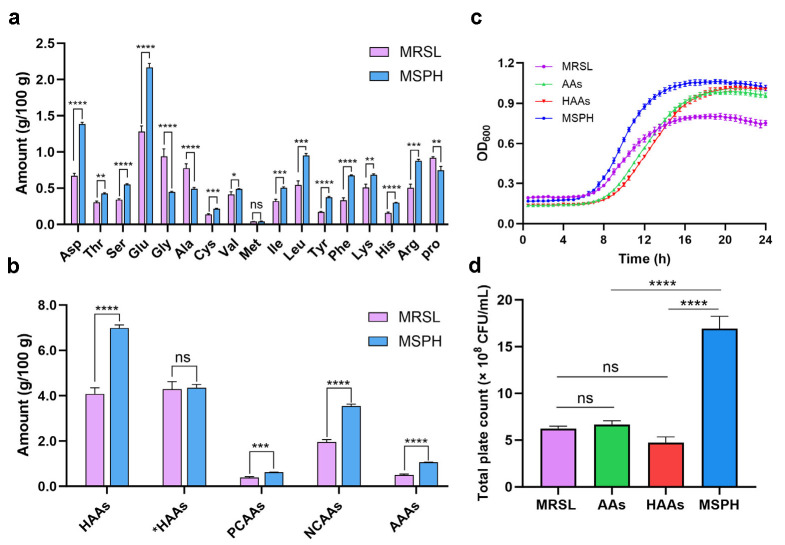
(**a**) Amino acid composition and (**b**) contents of amino acids by category in MRSL and MSPH medium. Hydrophilic amino acids (HAAs): Ser, Thr, Cys, Asp, Tyr, Glu, Tyr, Lys, Arg, His; hydrophobic amino acids (*HAAs): Ala, Val, Ile, Leu, Phe, Pro, Met, Gly; positively charged amino acids (PCAAs): Arg, His, Lys; negatively charged amino acids (NCAAs): Asp, Glu; aromatic amino acids (AAAs): Phe, Tyr; (**c**) growth curves; and (**d**) total plate count at 24 h for *B. breve* 1206 cultured in amino acids (AAs) and HAAs medium, in comparation with MRSL and MSPH. Statistical significance was evaluated using GraphPad Prism. The number of asterisks denotes the statistical significance level. ns indicates no significant difference, * indicates *p* < 0.05, ** indicates *p* < 0.01, *** indicates *p* < 0.001, and **** indicates *p* < 0.0001.

As SPH at different substitution levels all positively influenced the growth and viability of the *B. breve* 1206 strain. The amino acid profiles of the fully nitrogen-source-substituted MSPH and MRSL media were analyzed. As shown in Figure 3a, both the MRSL and MSPH medium provided essential amino acids for the growth of *B. breve* 1206. It can be observed that the contents of the 17 types of amino acids in MRSL and MSPH show significant differences, except for methionine (Met). For hydrophilic amino acids (HAAs), the levels of aspartic acid (Asp) (*p* < 0.0001), threonine (Thr) (*p* < 0.01), serine (Ser) (*p* < 0.0001), glutamic acid (Glu) (*p* < 0.0001), cysteine (Cys) (*p* < 0.001), lysine (Lys) (*p* < 0.01), histidine (His) (*p* < 0.0001), and arginine (Arg) (*p* < 0.001) in MSPH were significantly higher than those in MRSL. Peptides rich in these residues or with high hydrophilicity were found to strongly enhance the growth of *Bifidobacterium* and *Lactobacillus* [10,41,42]. Notably, the sulfhydryl groups were abundant in MSPH due to its higher Cys content of SPH, and these groups were known to be critical for stimulating *Bifidobacterium* growth [43]. The contents of hydrophobic amino acids (*HAAs), including valine (Val) (*p* < 0.05), isoleucine (Ile) (*p* < 0.001), leucine (Leu) (*p* < 0.001), tyrosine (Tyr) (*p* < 0.0001), and phenylalanine (Phe) (*p* < 0.0001), were significantly higher in MSPH, while the levels of glycine (Gly) (*p* < 0.0001), alanine (Ala) (*p* < 0.0001), and proline (Pro) (*p* < 0.01) were distinctly lower compared to MRSL. Previous studies showed that hydrolysates rich in amino acid residues of Ala, Met, Tyr, Phe, and Arg strongly stimulated *Bifidobacterium* growth, with Tyr being especially critical [44]. The contents of the critical hydrophilic amino acid residues (Asp, Thr, Ser, Glu, Cys, Lys, His, and Arg) and hydrophobic amino acid residues (Tyr and Phe) may explain the superior growth-promoting effects of MSPH.

Figure 3b illustrated the total contents of hydrophobic and hydrophilic of amino acids. A significant difference was observed in the total HAAs contents between the MSPH and MRSL (*p* < 0.0001), while the total *HAAs contents showed no significant variation. High content of hydrophilic amino acids may be the key factor stimulating the growth of *B. breve* 1206. This observation was consistent with findings by Zhao et al. [39]. They found that SPH could promote the growth of *Saccharomyces cerevisiae* due to hydrophilic and positively charged amino acids, such as Lys, His, Arg, and Ile. Compared to MRSL, MSPH contained higher levels of positively charged amino acids (PCAAs), negatively charged amino acids (NCAAs), and aromatic amino acids (AAAs), which may have further contributed to its growth-promoting effects.

The results indicate that the growth-promoting effect of SPH was closely related to hydrophilic amino acid residues such as Asp, Thr, Ser, Glu, Cys, Lys, His and Arg, with hydrophobic amino acid residues like Tyr and Phe also contributing to its effect. It was evident that *B. breve* 1206 showed high complexity in the utilization of exogenous amino acids through the peptide transport system. Further studies should explore the effect of specific free amino acids or peptides composed of specific amino acid residues on the growth and characteristics of *Bifidobacterium.*

To verify whether the growth-promoting effect of SPH was attributed to specific free amino acids or to peptides composed of amino acid residues, nitrogen-equivalent amino acid (AAs) and hydrophilic amino acid (HAAs) media were prepared based on the amino acid composition of SPH and used as nitrogen sources. The growth of *B. breve* 1206 was measured and compared with that in MRSL and MSPH described above. The results showed that *B. breve* 1206 grown in the AAs and HAAs medium entered the logarithmic phase later than those in MRSL and MSPH. However, their OD_600_ values exceeded that of MRSL at around 14 h and approached that of MSPH at 24 h, indicating that free amino acids may also contribute to the growth-promoting effect (Figure 3c). Although the amino acid medium culture increased the OD_600_ value, the viable cell count did not show a significant difference from MRSL at 24 h (Figure 3d). And the viable cell counts in both AAs and HAAs were markedly lower than in MSPH (*p* < 0.0001). The reason for the contradiction between OD_600_ value and the viable cell count might be that the cells are prone to death in the AAs and HAAs culture environment or show poor tolerance to acidic conditions. Thus, it can be seen that the peptide composed of specific amino acid residues is the key factor for promoting growth, rather than the amino acids themselves.

### 3.4. Morphology Analysis of MRSL and MSPH

**Figure 4:**
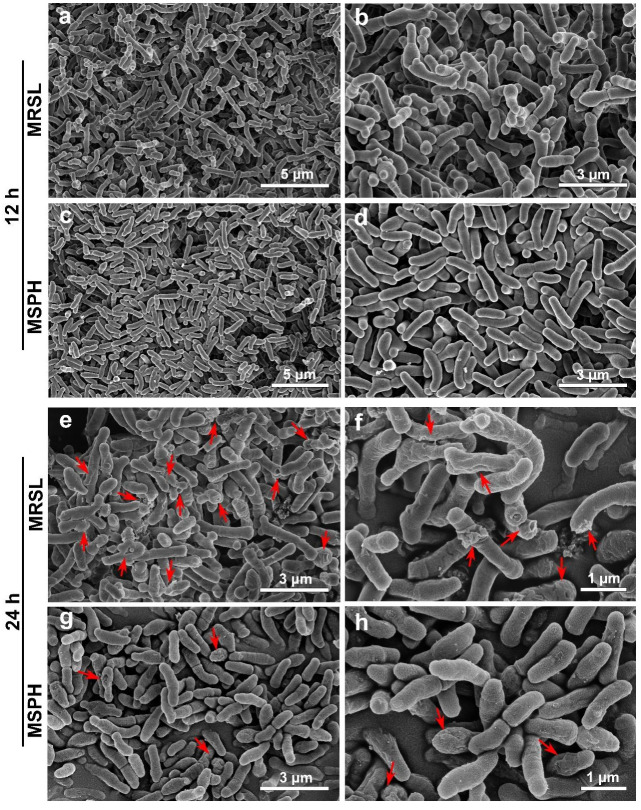
The SEM image of the *B. breve* 1206 cultured in MRSL and MSPH medium for (**a**–**d**) 12 h and (**e**–**h**) 24 h. Damaged regions of bacterial cells are marked by red arrows in SEM images.

The SEM results showed that *Bifidobacterium* cells appeared in both rod and Y shapes cultured in MRSL cultivation for 12 h (Figure 4a,b). In contrast, the cells cultured in MSPH medium mainly exhibited rod-shaped cells, with a notable reduction in Y forms (Figure 4c,d). Previous study suggested that *Bifidobacterium* tended to exhibit highly branched forms under unfavorable growth conditions, whereas rod-shaped cells dominated when adapting to the cultivation environment and were more oxygen-tolerant [45]. This may be related to the significantly higher levels of glutamic acid (Glu), aspartic acid (Asp), and serine (Ser) in MSPH compared to MRSL (Figure 3a). These three amino acids, along with alanine (Ala), were considered to be the critical amino acids for the conversion of the branched form into the more stable rod shape [46]. Therefore, the Y-to-rod transition of *B. breve* 1206 observed under the 100%-SPH condition is likely to reflect a nutrient-supported adaptive change in cell shape, although the precise molecular mechanism remains to be clarified and will require further investigation. Moreover, after 24 h of MRSL incubation, the wrinkles and visible broken fragments of cells appeared (Figure 4e,f). In contrast, MSPH cells were plump and round with less damage (Figure 4g,h). This indicated that MSPH facilitated the formation of a more stable rod-shaped morphology in *B. breve* 1206 and maintained its integrity in acidic environments, resulting in better growth and higher viable cell counts.

Previous study has indicated that the addition of calcium ions to the medium can induce morphological changes in lactic acid bacteria, thus enhancing their freeze tolerance [47]. Additionally, the hydrolysates of silver carp byproducts significantly altered the morphology of *Bifidobacterium animalis* ssp. *lactis* BB-12 during the logarithmic phase, resulting in shorter and thinner cells, which may facilitate accelerated division [48]. Similarly, shorter rod forms of *Limosilactobacillus reuteri* LR08 have been reported to promote growth by enabling faster binary fission [16]. These findings suggest that bacterial morphological changes may be related to their growth rate.

Overall, the SPH in MSPH medium provides a more favorable nitrogen source than the nitrogen source composed of bee extract, tryptone, and yeast extract in MRSL for the growth of *B. breve* 1206. The difference in nitrogen sources may affect bacterial growth and activity by inducing morphological changes.

### 3.5. CLSM Analysis of Live/Dead Bacteria in MRSL and MSPH

**Figure 5:**
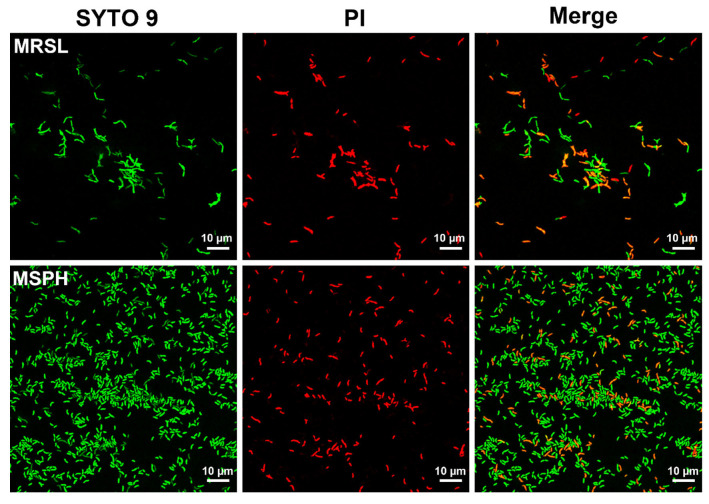
The cell viability of *B. breve* 1206 cultured in MRSL and MSPH for 24 h using confocal laser scanning microscopy. The live cells are green-stained with SYTO 9, while dead cells are red-stained with PI.

As shown in Figure 5, the results of confocal laser scanning microscopy showed that the morphology and damage of bacterial cells were consistent with the results of SEM. The Y- and rod-shaped cells of *B. breve* predominated when cultured in MRSL medium, while the rod-form cells were characterized in the MSPH medium. A large proportion of dead bacteria with red color was seen in the MRSL group, whereas the majority of bacteria cells were alive with green color in the MSPH group. The results suggested better cell wall integrity and bacterial viability in the MSPH group. A previous study reported that the components of medium, such as adding unsaturated fatty acids, can regulate cell membrane structure and improve freeze-drying survival rates [49]. In contrast to mixed nitrogen sources, the SPH in MSPH medium may facilitate the formation of a more stress-resistant cell membrane structure, resulting in protecting membrane integrity.

In addition, the lengths of dispersed bacteria were observed to be longer in MRSL than those in MSPH. Rajab et al. [50] first explored the influence of cell size of *Lactobacillus* strains on their probiotic properties and found that short *Lactobacillus* strains had higher CFU yield and stronger growth ability, while longer ones were more sensitive to acid and bile salts, resulting in lower CFU production.

### 3.6. Morphological Changes and Survival Rate After Freeze-Drying

**Figure 6:**
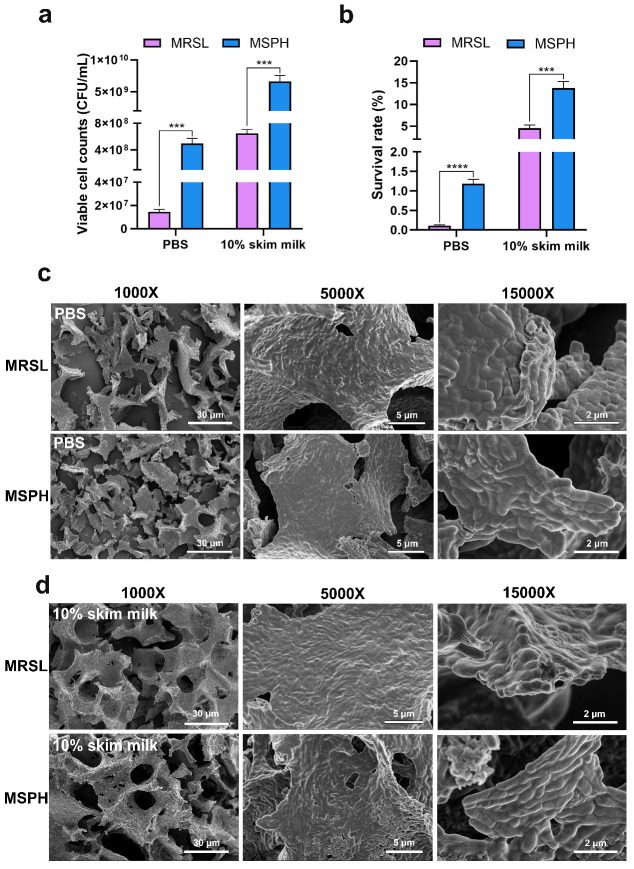
The effect of different nitrogen sources (MRSL and MSPH) on the (**a**) viable cell number; (**b**) survival rate; and scanning electron microscopy of freeze-dried *B. breve* 1206 with (**c**) PBS and (**d**) 10% skim milk as protectant. The number of asterisks denotes the statistical significance level based on GraphPad Prism analysis. *** indicates *p* < 0.001, and **** indicates *p* < 0.0001.

As shown in Figure 6a,b, the survival rate of *B. breve* 1206 after MRSL cultivation with 10% skim milk as the protectant was 4.53 ± 0.72%. In contrast, the survival rate significantly increased to 13.81 ± 1.53% after MSPH cultivation (*p* < 0.001), corresponding to a viable cell count of 6.60 ± 0.98 × 10^9^ CFU/mL. The number of viable bacteria was ten times higher than 6.47 ± 0.57 × 10^8^ CFU/mL in MRSL.

To exclude the potential effect of the freeze-drying protectant, PBS was also used as a protectant for further verification. The results showed that the survival rate of freeze-drying cells in MRSL cultivation was 0.14 ± 0.03%, while the survival rate in MSPH cultivation was 1.22 ± 0.10%, representing an 8.7-fold increase. These findings suggested that MSPH enhanced the freeze-drying tolerance of *B. breve* 1206 during the freeze-drying process.

The results of SEM images showed that bacterial cells cultured in MRSL and MSPH medium exhibited varying degrees of morphological changes and damage after lyophilization (Figure 6c,d). The PBS group exhibited more severe bacterial aggregation, distortion, and breakage than the 10% skim milk group. Furthermore, the bacterial structural integrity of the MSPH group was better than that of the MRSL group, leading to less deformation and fewer ruptures in both the PBS group and the 10% skim milk group.

Studies have suggested that the enhancement of freeze tolerance is associated with cell morphology and structure. Short rod-shaped lactobacilli showed lower mortality and damage rates during freezing storage compared to long filamentous cells [23,47]. Smaller cells contained less intracellular free water, which significantly improved the survival rate of *L. plantarum* IDCC 3501 after freeze-drying and subsequent storage [51]. The composition of the medium and the availability of nutrients are closely related to the changes in cell size and shape. In industrial production processes, the strategy of regulating the size and shape of *L. plantarum* B21 by controlling nutrient levels can produce functional probiotics with strong viability [52].

This also explains why MSPH has a higher freeze-drying survival rate than MRSL. Cultivation with MSPH induces the formation of smaller rod-shaped cells that exhibit better membrane integrity and increased resistance to adverse conditions such as water loss, temperature fluctuations, and oxidative damage during the lyophilization process.

### 3.7. Effects of MSPH on Bacterial Viability, Freeze-Drying Survival, and Morphology Changes in B. longum 070103 and 050101

**Figure 7:**
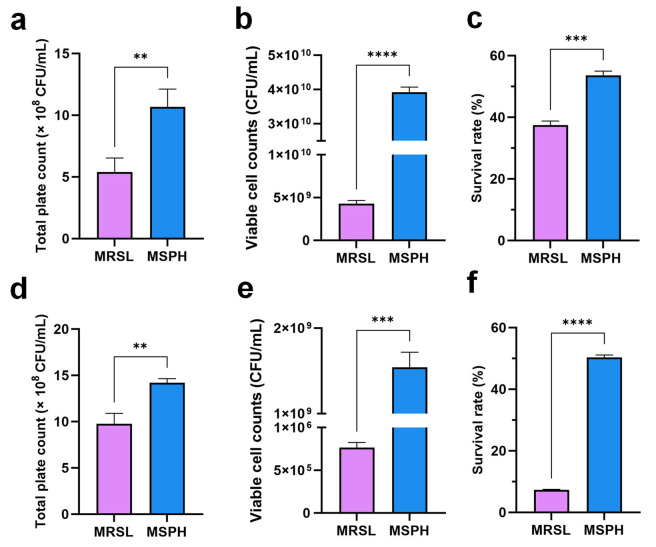
(**a**) Total plate count after 24 h cultivation in MRSL and MSPH; (**b**) viable cell count and (**c**) freeze-drying survival rate after cultivation with 10% skim milk for *B. longum* 070103. (**d**) Total plate count after 24 h cultivation in MRSL and MSPH; (**e**) viable cell count and (**f**) freeze-drying survival rate after cultivation with 10% skim milk for *B. longum* 050101. ** indicates *p* < 0.01, *** indicates *p* < 0.001, and **** indicates *p* < 0.0001.

The cell counts and freeze-drying tolerance of *B. breve* 1206 were enhanced by using SPH as the single nitrogen source. MSPH was also evaluated for its effect on bacterial viability and freeze-drying survival on the functional strains *B. longum* 070103 and 050101, previously developed by our team for health benefits [53]. The results indicate that SPH has a similar enhancing effect on *B. longum* 070103 and 050101 as it does on *B. breve* 1206. SPH significantly promoted the growth of functional *B. longum* 070103 (Figure 7a) and 050101 (Figure 7d). In the MSPH medium, both *B. longum* strains showed a significant increase in viable counts compared to the MRSL control group (*p* < 0.01). With 10% skim milk as a cryoprotectant, the viable count of *B. longum* 070103 after freeze-drying in MSPH was 3.92 × 10^10^ CFU/mL (hereinafter referred to as the lyophilization preservative volume), a tenfold increase compared to 4.28 × 10^9^ CFU/mL in the MRSL group (*p* < 0.0001). Similarly, *B. longum* 050101 had a viable count of 7.67 × 10^5^ CFU/mL in the MRSL group, which increased significantly to 1.54 × 10^9^ CFU/mL in MSPH (*p* < 0.001). The freeze-drying survival rates of *B. longum* 070103 and 050101 increased by approximately 16.2% (Figure 7c) and 43.1% (Figure 7f), respectively.

**Figure 8:**
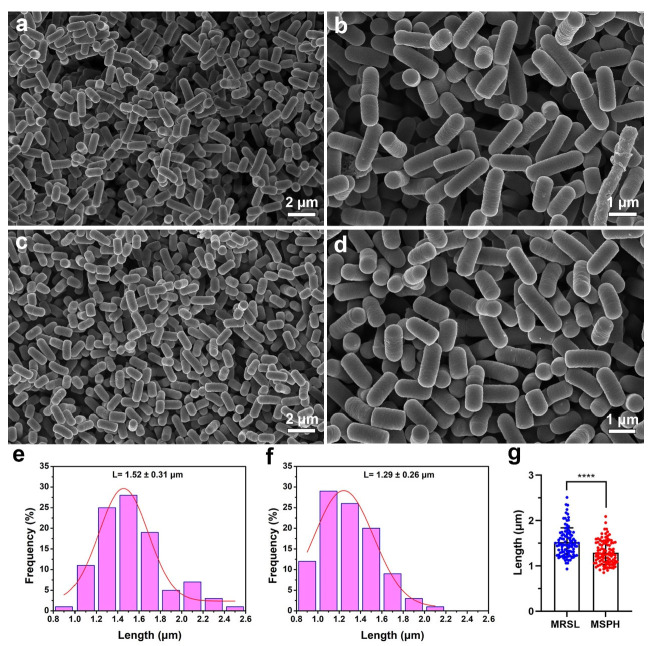
SEM images of *B. longum* 070103 after 24 h cultivation in (**a**–**b**) MRSL and (**c**–**d**) MSPH. Length distribution of 100 bacteria measured in (**e**) MRSL and (**f**) MSPH using Nano Measurer software; (**g**) statistical comparison of length distribution between MRSL and MSPH. **** indicates *p* < 0.0001.

**Figure 9:**
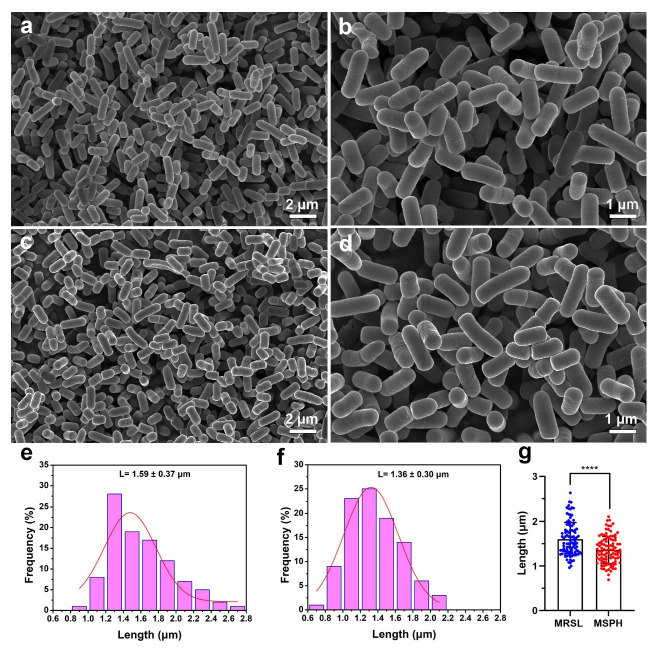
SEM images of *B. longum* 050101 after 24 h cultivation in (**a**,**b**) MRSL and (**c**,**d**) MSPH. Length distribution of 100 bacteria measured in (**e**) MRSL and (**f**) MSPH using Nano Measurer software; (**g**) statistical comparison of length distribution between MRSL and MSPH. **** indicates *p* < 0.0001.

Both *B. longum* strains were rod-shaped after 24 h of cultivation using SEM (Figure 8 and Figure 9). Nano Measurer software (version 1.2) was used for cell length analysis. The lengths of 100 bacteria cells cultured in MRSL (Figure 8a and Figure 9a) and MSPH (Figure 8c and Figure 9c) were measured, and their length distributions were plotted. Results showed that *B. longum* 070103 had an average length of 1.52 ± 0.31 μm in MRSL, which decreased to 1.29 ± 0.26 μm in MSPH (*p* < 0.0001, Figure 8g). Similarly, the average length of *B. longum* 050101 decreased from 1.59 ± 0.37 μm in MRSL to 1.36 ± 0.30 μm in MSPH (*p* < 0.0001, Figure 9g). These results demonstrated that MSPH induced smaller or shorter cells, which may improve membrane integrity and resistance to freeze-drying stress.

These findings highlight the potential application of soy protein hydrolysates as a sole nitrogen source to promote growth and freeze-drying survival of *Bifidobacterium*, demonstrating its potential for industrial use. Furthermore, optimizing culture media to regulate probiotic morphology is a crucial strategy for selecting strains with strong fermentation capacity and freeze-drying tolerance. A limitation of the present work is that the long-term stability and morphological integrity of SPH-cultured strains in real commercial food matrices (e.g., fermented dairy products, milk powders, or dietary supplements) were not investigated. Future work should clarify whether the resistance induced by SPH supports sustained viability during long-term storage in complex food matrices.

## 4. Conclusions

This study demonstrated that SPH can serve as an effective sole nitrogen source for the cultivation of functional *B. breve* and *B. longum* strains, improving both their growth and freeze-drying survival. These effects are likely related to the high content of low-molecular-weight peptides and rich hydrophilic amino acid residues in SPH. Furthermore, SPH induced a morphological change toward shorter rod-shaped cells. This morphology helped to maintain cell membrane integrity in acidic conditions and the freeze-drying process, resulting in an increased viable cell number and enhanced freeze-drying resistance. These results indicate that the composition of peptides and bacterial morphology can be regulated through nitrogen sources, thereby enhancing the growth efficiency and environmental tolerance of probiotics. Overall, this study supports the potential use of plant-derived soybean peptides as nitrogen sources for high-density fermentation, freeze-drying storage, and subsequent applications of functional probiotics, as well as their potential as prebiotics with health benefits.

## Data Availability

The original contributions presented in the study are included in the article; further inquiries can be directed to the corresponding author.
